# [^161^Tb]Tb-BPAMD as a High-Affinity Agent for Skeletal Targeting: Radiochemical and Biodistribution Insights

**DOI:** 10.3390/pharmaceutics18030312

**Published:** 2026-02-28

**Authors:** Magdalena Radović, Pavle Sitarica, Dragana Stanković, Marija Mirković, Drina Janković, Miloš Marić, Marko Perić, Sanja Vranješ-Đurić, Aleksandar Vukadinović

**Affiliations:** “VINČA” Institute of Nuclear Sciences, National Institute of the Republic of Serbia, University of Belgrade, 11001 Belgrade, Serbia; pavle.sitarica@vin.bg.ac.rs (P.S.);

**Keywords:** BPAMD, [^161^Tb]Tb-bisphosphonates, hydroxyapatite binding, biodistribution, bone-targeting, DFT

## Abstract

**Background**: Bone-seeking radiopharmaceuticals based on bisphosphonates enable targeted therapy of skeletal metastases. They are suitable carriers for therapeutic radionuclides such as terbium-161 (^161^Tb), a β^−^ emitter that additionally releases short-range conversion and Auger electrons, which may enhance radiation dose delivery to small lesions. This study explored the potential of the well-established DOTA conjugated bisphosphonate BPAMD (4-{[(bis(phosphonomethyl))carbamoyl]methyl}-7,10-bis(carboxymethyl)-1,4,7,10 tetraazacyclododec-1-yl)acetic acid) radiolabeled with ^161^Tb as a bone-targeted radiopharmaceutical, focusing on the theranostic and radiophysical advantages conferred by the radionuclide. **Methods**: BPAMD was radiolabeled with ^161^Tb and ^177^Lu under mild conditions (pH 4.5, 95 °C, 30 min); subsequently, the radiochemical purity was assessed by radio-TLC. Physicochemical properties (charge, lipophilicity, protein binding), in vitro stability (saline and human serum, 48 h), and hydroxyapatite (HAP) binding were evaluated for [^161^Tb]Tb-BPAMD. Biodistribution was investigated in healthy Wistar rats (*n* = 3 per time point) at 2 h, 24 h, and 7 days post-injection. Computational density functional theory (DFT) analyses were performed to explore the coordination chemistry of Tb^3+^ and Lu^3+^ with BPAMD. **Results**: Both complexes achieved a radiochemical yield of greater than 98%. [^161^Tb]Tb-BPAMD exhibited negative charge, high hydrophilicity (logP = −3.92 ± 0.13), low protein binding (19.07 ± 1.01%), excellent radiochemical stability under simulated physiological conditions (>97% at 48 h), and strong hydroxyapatite affinity (>98% with ≥10 mg HAP). Biodistribution showed high, stable bone uptake (8.06% ID/g at 2 h; 6.70% ID/g at 24 h; 5.31% ID/g at 7 d) with rapid blood clearance (<0.001% ID/g at 24 h) and low non-target retention. To contextualize its performance, [^161^Tb]Tb-BPAMD was compared with [^177^Lu]Lu-BPAMD, which demonstrated similarly strong skeletal retention (8.74% ID/g at 2 h; 8.08% ID/g at 24 h; 5.25% ID/g at 7 d) but comparatively higher non-target organ uptake. DFT calculations indicate that both Tb^3+^ and Lu^3+^ favor octa-coordinated BPAMD complexes. **Conclusions**: [^161^Tb]Tb-BPAMD exhibits excellent radiochemical and pharmacokinetic properties, with enhanced biodistribution selectivity over [^177^Lu]Lu-BPAMD. Combined with the radiobiological advantages of ^161^Tb, it represents a promising theranostic candidate for targeted therapy of bone metastases.

## 1. Introduction

Bone metastases are a frequent and serious complication of advanced cancers, particularly those originating from the prostate, breast, and lungs [1,2,3]. These lesions are associated with significant morbidity, including intractable pain, pathological fractures, spinal cord compression, and hypercalcemia, which collectively reduce patient quality of life and increase healthcare burden [4,5,6]. Radiopharmaceutical therapy targeting skeletal metastases has emerged as a valuable strategy for palliative care, offering site-specific irradiation with minimal systemic toxicity [7,8,9].

Among the established radiotherapeutics, β^−^-emitting agents such as [^153^Sm]Sm-EDTMP and [^177^Lu]Lu-EDTMP have been employed for decades, demonstrating clinical efficacy [10,11,12,13]. However, these first-generation polyphosphonate-based agents exhibit some limitations in terms of in vivo stability and the need for high ligand concentrations due to weaker metal–ligand binding affinity [14,15,16,17]. The reduced kinetic stability and relatively modest specific activity of [^177^Lu]Lu-EDTMP could result in suboptimal bone targeting [14,15].

To address these drawbacks, the macrocyclic chelator DOTA has been conjugated with a bisphosphonate to form BPAMD, a well-defined bone-targeting molecule. This molecule combines the strong metal-chelating properties of DOTA with a bisphosphonate moiety, which exhibits high affinity for hydroxyapatite, the primary mineral component of bone. Radiolabeled with ^177^Lu, it demonstrated high radiolabeling yields, excellent in vitro and in vivo stability, and outstanding bone uptake [14,18,19]. Moreover, the DOTA-based design of BPAMD allows it to function as a theranostic agent [15,20]. Gallium-68-labeled BPAMD has been successfully used for PET imaging of skeletal metastases, enabling precise localization and dosimetry before the therapeutic administration of the corresponding ^177^Lu-labeled compound [21]. In early human studies, [^177^Lu]Lu-BPAMD showed strong accumulation in osteoblastic lesions and minimal uptake in soft tissues, with a favorable toxicity profile and significant pain reduction [22]. Beyond ^177^Lu, BPAMD has also been successfully radiolabeled with other therapeutic β-emitting radionuclides, including ^90^Y, ^175^Yb, and ^153^Sm, for the palliative management of painful bone metastases, further demonstrating the adaptability and broad therapeutic potential of this bisphosphonate-based ligand in radionuclide therapy [23,24,25,26,27]. These findings collectively underscore the potential of macrocyclic bisphosphonates, such as BPAMD, to serve as adaptable and effective platforms for personalized treatment of skeletal metastatic disease [15].

In recent years, terbium-161 (^161^Tb) has attracted considerable interest as a therapeutic radionuclide due to its favorable physical and radiochemical characteristics [28,29,30,31]. Like ^177^Lu, ^161^Tb is a medium-energy β^−^ emitter with a similar physical half-life (~6.9 days) and energy profile [32]. However, ^161^Tb offers an additional advantage: it emits a substantial number of low-energy Auger and internal conversion electrons, which deposit high linear energy transfer (LET) radiation over nanometer to micrometer ranges, making it suitable for the treatment of small and micrometastatic lesions [33,34]. This enhanced LET is particularly beneficial for targeting small tumor clusters and isolated tumor cells, where conventional β^−^ emitters may fall short [35,36,37,38,39,40].

In addition to its therapeutic potential, ^161^Tb also emits low-energy γ-photons suitable for SPECT imaging, making it compatible with theranostic workflows [41,42,43,44,45]. It can be stably coordinated by DOTA, and previous studies have demonstrated the feasibility of radiolabeling DOTA-based molecules with terbium radionuclides [30,46]. Taken together, these properties position ^161^Tb as a compelling alternative to ^177^Lu, potentially offering enhanced therapeutic efficacy while retaining similar pharmacokinetics.

Despite these promising features, there is limited literature on ^161^Tb-labeled bisphosphonates. Given the proven performance of [^177^Lu]Lu-BPAMD and the additional cytotoxicity potential offered by ^161^Tb, it is logical to explore the substitution of ^161^Tb within a well-characterized bisphosphonate framework, focusing on the potential therapeutic advantages offered by the radionuclide itself. The structural compatibility of BPAMD with both ^177^Lu and ^161^Tb enables this approach without implying modifications in the ligand design or alterations in pharmacokinetic behavior.

This study aims to investigate the radiolabeling yield, in vitro stability, and biodistribution characteristics of [^161^Tb]Tb-BPAMD. Radiolabeling was performed under practical conditions suitable for potential clinical translation, and the radiocomplex stability was assessed in saline as well as in human serum. In vitro hydroxyapatite binding studies were conducted to confirm high mineral affinity, which is critical for effective skeletal targeting. Biodistribution experiments were performed in healthy Wistar rats to evaluate in vivo skeletal uptake and non-target tissue clearance. In parallel, [^177^Lu]Lu-BPAMD was prepared under the same experimental conditions to provide a reference framework using a clinically established β^−^-emitting therapeutic. To provide insights into the coordination chemistry of Tb and Lu complexes, DFT computational studies were performed. By systematically investigating these parameters, this study evaluates the potential of [^161^Tb]Tb-BPAMD as an improved bone-targeting therapeutic agent. If confirmed, [^161^Tb]Tb-BPAMD could provide enhanced radiation dose delivery at metastatic sites through the emission of short-range electrons, while retaining pharmacokinetics comparable to [^177^Lu]Lu-BPAMD, ultimately improving therapeutic efficacy and reducing systemic toxicity.

## 2. Materials and Methods

### 2.1. Chemicals and Reagents

The ligand (4-{[(bis(phosphonomethyl))carbamoyl]methyl}-7,10-bis(carboxymethyl)-1,4,7,10-tetraazacyclododec-1-yl) acetic acid (BPAMD) was purchased from ABX (Radeberg, Germany). BPAMD stock solution (1 mg/1 mL) was prepared in ultrapure water and used immediately before the experiments. All other chemical reagents, sourced from Merck KGaA (Darmstadt, Germany), were of analytical grade and used as received.

No-carrier-added [^161^Tb]TbCl_3_ solution in 0.05 M HCl was provided by Terthera, Breda, The Netherlands, in a volume of 500 µL containing activity of 1 GBq. No-carrier-added ^177^LuCl_3_ was supplied by ITG Isotope Technologies Garching GmbH (Munich, Germany) with a specific activity >500 GBq/mg Lu.

A freshly made 0.4 M sodium acetate buffer (pH 4.5) was prepared from sodium acetate trihydrate and glacial acetic acid of analytical-grade, with pH adjustments performed using 0.1 M HCl or NaOH. Ultrapure deionized water from a Milli-Q purification system (Millipore, Billerica, MA, USA) was used for all aqueous solutions.

### 2.2. Equipment

The radioactivity of the samples was measured using a gamma counter (WIZARD 2480, PerkinElmer, Shelton, CT, USA) and a dose calibrator (CRC-15R beta, Capintec Inc., Ramsey, NJ, USA). Radio-thin layer chromatography (radio-TLC) was carried out using a Scan-RAM scanner (LabLogic Group, Sheffield, South Yorkshire, UK).

### 2.3. Radiolabeling of BPAMD with ^177^Lu

The radiolabeling of BPAMD with ^177^Lu was performed according to the methods described by Meckel et al. (2015) and Bergmann et al. (2016) [15,18], with minor modifications to optimize reproducibility. Briefly, 74 MBq of [^177^Lu]LuCl_3_ was added in an aqueous solution containing 100 µg of BPAMD in 0.5 mL of sodium acetate buffer (0.4 M, pH 4.5). The reaction mixture was heated to 95 °C and maintained for 30 min. After heating, the reaction mixture was allowed to cool to room temperature. The resulting solution was directly analyzed by radio-TLC without any further purification.

### 2.4. Radiolabeling of BPAMD with ^161^Tb

The labeling of BPAMD with ^161^Tb was performed under the same conditions as those for ^177^Lu. A solution of 100 µg BPAMD in 0.5 mL acetate buffer (0.4 M, pH 4.5) was mixed with 74 MBq of ^161^TbCl_3_ and heated at 95 °C for 30 min in a sealed vial. After cooling to room temperature, the reaction mixture was analyzed by radio-TLC.

### 2.5. Radiochemical Purity Assessment

Radiochemical purity (RCP) was evaluated by radio-TLC using silica gel ITLC strips (ITLC-SG, Agilent Technologies, Santa Clara, CA, USA). In brief, 5 µL of the radiolabeled solution and a control sample of pure radionuclides were placed on the strips and developed with two solvent system: 1 M NaOH and ammonia/ethanol/water (1:10:20, *v*/*v*/*v*), in which the ammonia component was 25% *w*/*w* aqueous NH_3_ [23,24,25,26]. After development up to 12 cm, the strips were dried and scanned with a gamma/TLC scanner to determine the migration of radiolabeled complexes versus free radionuclides, providing an assessment of radiochemical purity. In the system, the radiolabeled complexes moved with the solvent front, whereas unbound radionuclides remained at the origin. RCP was calculated as the percentage of radioactivity associated with the complexes relative to total activity.

### 2.6. Radioelectrophoresis

The electrophoretic behavior of the [^161^Tb]Tb-BPAMD complex and the success of radiolabeling were examined by radioelectrophoresis. A 5 μL aliquot of the radiolabeled complex or a control sample of [^161^Tb]TbCl_3_ was spotted at the center of Whatman 3MM chromatography paper strips (2 × 25 cm), pre-soaked in 0.05 M phosphate buffer (pH 7.4). The strips were then placed in an electrophoresis chamber containing the same buffer and run at 250 V for 1 h using a Gelman Instrument electrophoresis system (USA). After completing the radioelectrophoresis, each strip was dried, and the distribution of radioactivity along the strip was then measured using a gamma/TLC scanner. This approach enabled a comparison of the migration behavior of [^161^Tb]Tb-BPAMD with free ^161^Tb^3+^, offering insight into their charge [47].

### 2.7. Determination of Lipophilicity

The partition coefficient (log P) of the [^161^Tb]Tb-BPAMD complex was determined using the shake-flask method [48]. Equal volumes (1 mL) of 1-octanol and phosphate-buffered saline (PBS, pH 7.4) were pre-equilibrated, and 100 µL of the radiolabeled complex was added. The samples were mixed by vortexing for 1 min and centrifuged at 3000 rpm for 5 min to allow phase separation. Radioactivity in both the organic and aqueous phases was determined with a gamma counter. Experiments were performed in triplicate, and log P was calculated as the logarithm of the ratio of counts in the octanol to aqueous phase.

### 2.8. Protein Binding Assay

The extent of protein binding of [^161^Tb]Tb-BPAMD was assessed using the trichloroacetic acid (TCA) precipitation method. A 100 µL aliquot of the radiolabeled complex was mixed with 500 µL of 12% (*w*/*v*) human serum albumin (HSA) solution (provided by the National Blood Transfusion Institute, Belgrade, Serbia) and incubated at 37 °C for 20 min. Protein-bound species were then precipitated by the addition of 1 mL of 20% (*w*/*v*) trichloroacetic acid (TCA). Following centrifugation at 3000 rpm for 5 min, the precipitate was washed with 0.9% NaCl to remove residual unbound activity. This washing step was repeated three times to allow complete separation of bound (precipitate) and unbound (supernatant) fractions. The radioactivity of fractions was quantified independently using a gamma counter. Experiments were performed in triplicate. The percentage of protein-bound [^161^Tb]Tb-BPAMD was calculated by comparing the radioactivity in the precipitate to the total sample radioactivity (precipitate + supernatant) and expressing it as a percentage.

### 2.9. Hydroxyapatite Binding Assay

In vitro hydroxyapatite (HAP) binding affinity was evaluated to assess bone-targeting potential [49]. Varying amounts (1, 2, 5, 10, 20, 30, 40, and 50 mg) of HAP powder were suspended in 2 mL of saline solution and pre-equilibrated by gentle shaking for 2 h. Subsequently, 50 µL of [^161^Tb]Tb-BPAMD was added and incubated for 24 h at room temperature. After centrifuging the samples, the radioactivity in both the precipitate and supernatant was determined. HAP binding was calculated as the fraction of total radioactivity present in the precipitate.

### 2.10. In Vitro Stability Studies

The in vitro stability of [^161^Tb]Tb-BPAMD was evaluated in saline and human serum at 37 °C over 48 h. Radiolabeled aliquots (100 µL, 14.8 MBq) were added to 900 µL of saline or human serum. Experiments were performed in triplicate. At defined time points (2, 24, and 48 h), samples were analyzed using the radio-TLC method described above to determine the percentage of intact complex versus free radioisotope.

### 2.11. Biodistribution Studies

Biodistribution experiments were carried out in healthy Wistar rats (100–120 g, *n* = 3 per time point) obtained from a colony of the Military Medical Academy (Belgrade, Serbia). Animals were kept under standard conditions (12 h light–dark cycle, 21 ± 2 °C, (40–45%) humidity, and 120 lx illumination). All animal procedures were approved by the Ethical Committee of the Vinča Institute of Nuclear Sciences (permission No. 002225501 2025, issued on 20 May 2025) and were in accordance with the Directive 2010/63/EU of the European Parliament and of the Council of 22 September 2010 [50]. Rats were injected intravenously via the tail vein with 0.1 mL of [^177^Lu]Lu-BPAMD or [^161^Tb]Tb-BPAMD (~0.74 MBq). At 2 h, 24 h, and 7 d after injection, animals were sacrificed, and selected organs (blood, heart, lungs, liver, spleen, kidneys, stomach, intestines, muscle, and femur) were collected, weighed, and measured for radioactivity using a gamma counter. The biodistribution results were exported as a percentage of injected dose per gram of tissue (%ID/g) and presented as mean ± SD (*n* = 3).

### 2.12. Computational Details

Geometry optimizations and energy minimizations were performed using the BP86 level of theory [51]. For the heavy lanthanide Tb^3+^ ion, SARC2-DKH-QZVP basis set has been applied, and for all other atoms, DKH-def2-SVP basis set has been used [52]. Additionally, the SARC/J auxiliary basis set, a decontracted version of the def2/J set, was employed to enhance accuracy in the relativistic calculations [53,54,55,56]. All calculations were performed using the ORCA program system, version 6.0 [57,58,59,60,61].

## 3. Results and Discussion

### 3.1. Radiolabeling Yield and Radioelectrophoresis

**Scheme 1:**
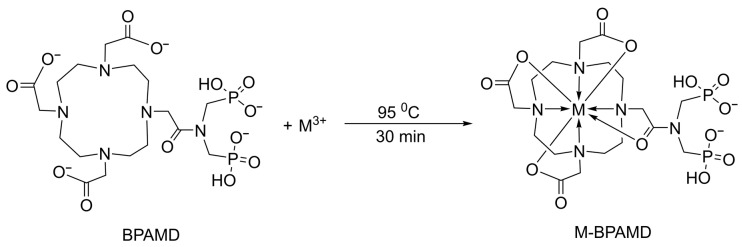
Synthesis of M-BPAMD complexes (M = Tb, Lu).

Radiolabeling of BPAMD with ^161^Tb and ^177^Lu was successfully performed under identical conditions, mildly acidic pH (4.5) and elevated temperature (95 °C) for 30 min, resulting in high radiolabeling yields exceeding 98% for both radionuclides. The complex-formation reaction depicted in Scheme 1 illustrates the chelation of the metal center by the BPAMD ligand. Computational analysis, using DFT, indicates that both metals favor octa-coordination over hepta-coordination, as explained in the Supplementary Materials.

**Figure 1:**
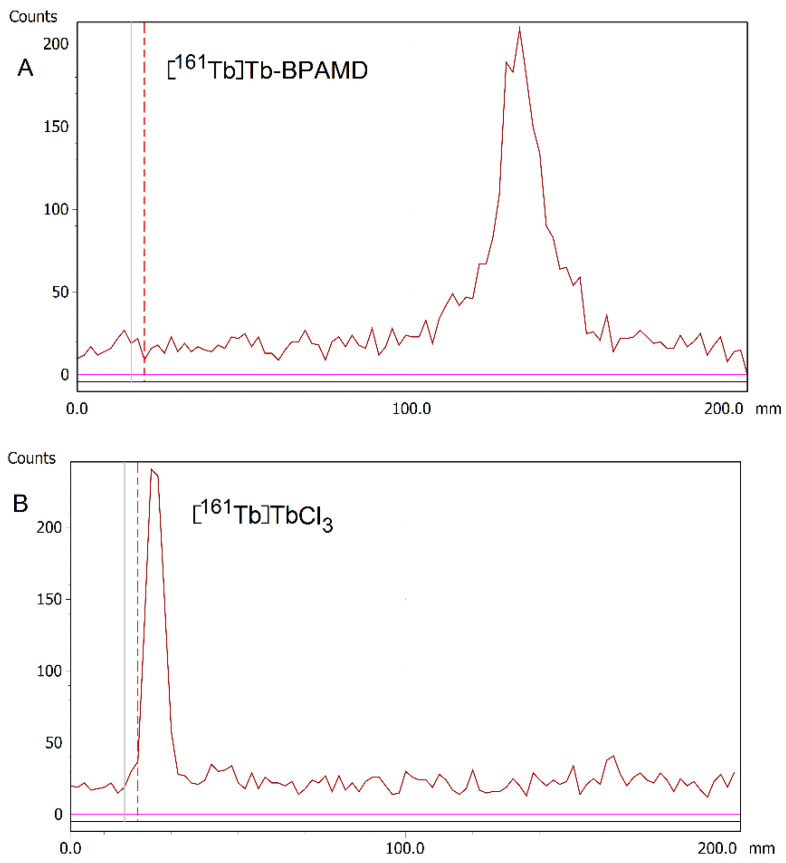
Radio-TLC chromatograms of [^161^Tb]Tb-BPAMD (**A**) and [^161^Tb]TbCl_3_ (**B**).

The radiolabeling yield for [^177^Lu]Lu-BPAMD agrees with previously reported values for analogous DOTA-conjugated bisphosphonates radiolabeled with ^177^Lu or ^68^Ga [18,62,63]. Owing to the high incorporation efficiency and negligible presence of unbound ^161^TbCl_3_, post-labeling purification was not required, simplifying the preparation process for further in vitro and in vivo applications. Further experiments were carried out only for [^161^Tb]Tb-BPAMD, whereas [^177^Lu]Lu-BPAMD was included only in the biodistribution study in healthy Wistar rats to enable direct comparison with the ^161^Tb-labeled analogue. The representative radio-TLC chromatograms of [^161^Tb]Tb-BPAMD and free [^161^Tb]TbCl_3_ obtained using ammonia/ethanol/water (1:10:20, *v*/*v*/*v*; 25% *w*/*w* NH_3_) as the primary mobile phase are shown in Figure 1. As a complementary approach, 1 M NaOH was applied to qualitatively distinguish between complexed and free radionuclides. In both systems, [^161^Tb]Tb-BPAMD consistently moved with the solvent front, while free [^161^Tb]TbCl_3_ stayed at the origin, indicating efficient chelation and absence of detectable unchelated species. Radio-TLC chromatograms for both mobile phases are provided in the Supplementary Material (Figures S1–S4).

**Figure 2:**
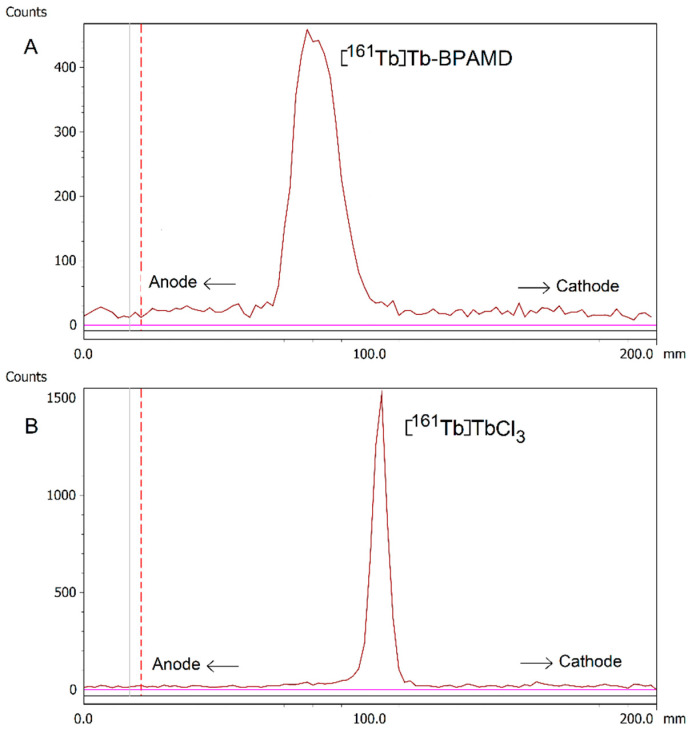
Radioelectrophoresis analysis of [^161^Tb]Tb-BPAMD (**A**) and [^161^Tb]TbCl_3_ (**B**).

The electrophoretic behavior of the complex was determined by radioelectrophoresis, performed at physiological pH (7.4). Under these conditions, [^161^Tb]Tb-BPAMD migrated toward the positively charged anode, indicating a negatively charged complex nature (Figure 2A). In contrast, free [^161^Tb]TbCl_3_ remained at the origin (Rf = 0), indicating neutral or colloidal hydrolyzed terbium species (Figure 2B). These findings confirm the effective chelation of terbium(III) by BPAMD and the absence of any detectable colloidal aggregates or hydrolysis products under physiological pH and ionic strength conditions. The observed electrophoretic behavior is a relevant characteristic for bone-seeking radiopharmaceuticals, as charge-related properties influence plasma protein interactions, renal clearance, and non-target organ uptake. In the case of [^161^Tb]Tb-BPAMD, the negative charge corresponds to high hydrophilicity and supports selective skeletal accumulation.

### 3.2. Protein Binding and Lipophilicity

The plasma protein binding value for [^161^Tb]Tb-BPAMD was determined to be 19.07 ± 1.01%, indicating a low affinity toward plasma proteins. While increased plasma protein binding, particularly through albumin-binding motifs, has been intentionally exploited in targeted radionuclide therapy to prolong blood circulation time and enhance tumor uptake [64], BPAMD lacks such modifications, and its low protein association reflects the inherent physicochemical properties of the parent bisphosphonate structure. This feature is expected to facilitate rapid systemic clearance and limit hepatic uptake, thereby reducing non-target radiation exposure and supporting a predictable pharmacokinetic profile for skeletal applications [22,65,66]. Furthermore, the measured log P of −3.92 ± 0.13 confirms the complex’s high hydrophilicity, consistent with other macrocyclic bisphosphonates [15,67]. This physicochemical profile suggests predominant renal excretion and minimal penetration across lipid-rich biological barriers. For bone-seeking radiopharmaceuticals intended for palliative therapy of skeletal metastases, rapid blood clearance combined with selective bone accumulation is often desirable. This pharmacokinetic profile yields high bone-to-soft tissue ratios and minimizes radiation exposure to non-target organs [15,68]. Consequently, the combination of low protein binding and high hydrophilicity supports the potential of [^161^Tb]Tb-BPAMD as a selective skeletal radiotherapeutic agent.

### 3.3. Hydroxyapatite Binding

**Figure 3:**
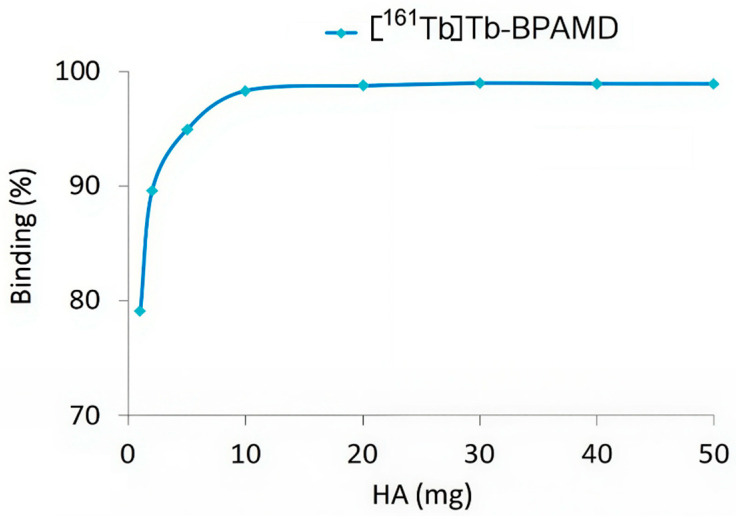
Hydroxyapatite binding of [^161^Tb]Tb-BPAMD complex after 24 h incubation at different HA amounts.

Hydroxyapatite (HA) binding studies of [^161^Tb]Tb-BPAMD revealed high-affinity, concentration-dependent interaction following 24 h incubation with increasing HA quantities (1–50 mg). Binding efficiency exceeded 98% at HA concentrations ≥10 mg (Figure 3), indicating rapid saturation. This profile closely parallels that of [^177^Lu]Lu-BPAMD, which similarly reached a binding plateau at relatively low HA levels [49]. The rapid saturation indicates that the bisphosphonate moieties within BPAMD possess sufficient affinity to achieve near-maximal binding even under conditions of limited mineral content, such as in early-stage osteoblastic lesions.

These results are consistent with known properties of bisphosphonate-based radiopharmaceuticals, which exhibit strong chemisorption to calcium phosphate surfaces through their phosphonate groups [69,70,71]. Effective skeletal targeting requires a high HAP binding capacity, particularly when minimizing uptake in non-calcified tissues and delivering therapeutic radiation doses to bone metastases.

### 3.4. In Vitro Stability

**Figure 4:**
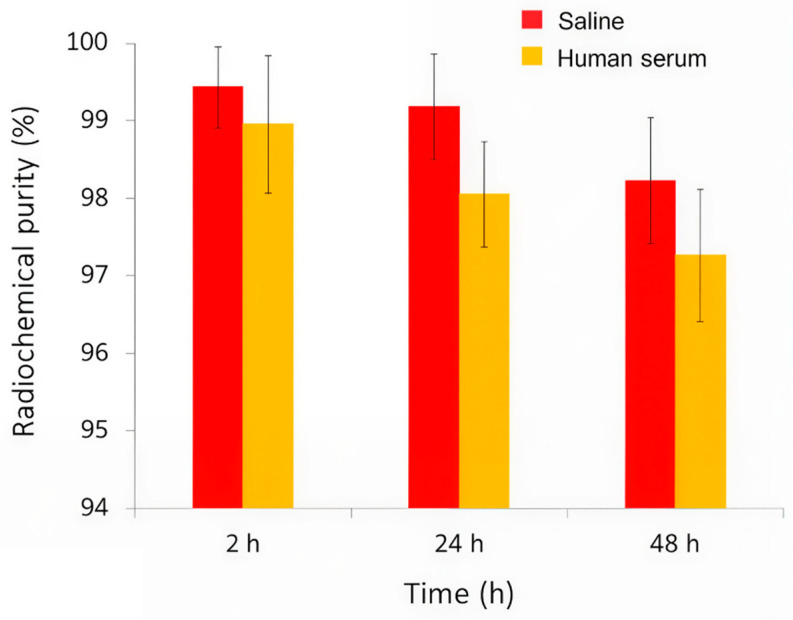
In vitro stability of [^161^Tb]Tb-BPAMD in saline and human serum at 37 °C.

The radiolabeled complex [^161^Tb]Tb-BPAMD was tested for in vitro stability in physiological saline and human serum at 37 °C over a period of 48 h (Figure 4). The results demonstrated that the complex exhibits excellent radiochemical stability under simulated physiological conditions. In saline, radiochemical purity remained remarkably high throughout the entire observation period, with values of 99.43 ± 0.52% at 2 h, 99.18 ± 0.68% at 24 h, and 98.22 ± 0.81% at 48 h, indicating negligible degradation or dissociation of the complex. Similarly, in human serum, where biological components such as proteins and ions could potentially interfere with the stability of the radiometal complex, [^161^Tb]Tb-BPAMD maintained high integrity, with 98.95 ± 1.09% at 2 h, 98.05 ± 0.68% at 24 h, and 97.26 ± 0.85% at 48 h. Although radiochemical purity showed a minor decrease over time in serum, it remained consistently above 97%, confirming that the complex resists transchelation and remains stable in the presence of endogenous biomolecules. This slight decrease is unlikely to be clinically significant, as the radiopharmaceutical’s uptake into bone is nearly complete within the first few hours post-injection, before any substantial degradation occurs. These findings match previous results for [^177^Lu]Lu-BPAMD and other DOTA-chelated bisphosphonates [49], and confirm that [^161^Tb]Tb-BPAMD is highly stable in vitro, supporting its potential for further in vivo evaluation and application in bone-targeted radionuclide therapy.

### 3.5. Biodistribution Studies of [^161^Tb]Tb-BPAMD and [^177^Lu]Lu-BPAMD

**Figure 5:**
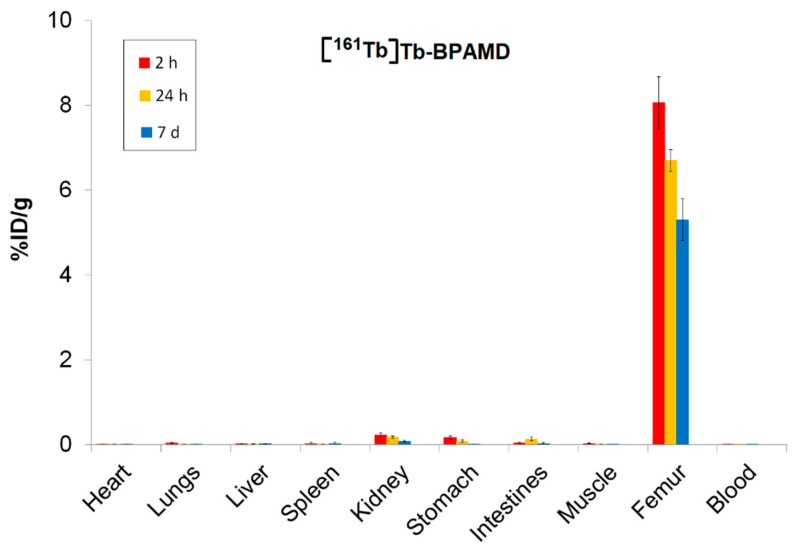
Biodistribution of [^161^Tb]Tb-BPAMD in healthy Wistar rats at 2 h, 24 h, and 7 d. Results are shown as mean ± SD (*n* = 3). %ID/g = indicates the percentage of injected dose per gram of tissue.

**Figure 6:**
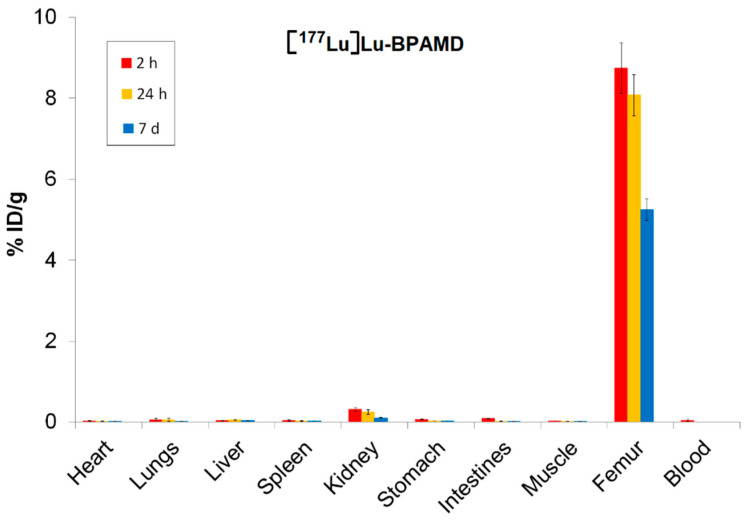
Biodistribution of [^177^Lu]Lu-BPAMD in healthy Wistar rats at 2 h, 24 h, and 7 d. Results are shown as mean ± SD (*n* = 3). %ID/g = indicates the percentage of injected dose per gram of tissue.

Biodistribution analysis of [^161^Tb]Tb-BPAMD and [^177^Lu]Lu-BPAMD in healthy Wistar rats provides insights into the therapeutic potential of both radiopharmaceuticals. For [^161^Tb]Tb-BPAMD (Figure 5), bone uptake remained high across all measured time points (8.06 ± 0.61%ID/g at 2 h; 6.70 ± 0.26%ID/g at 24 h; 5.31 ± 0.49%ID/g at 7 days), demonstrating stable skeletal retention. A comparable pattern was observed for [^177^Lu]Lu-BPAMD (Figure 6), which exhibited slightly higher early bone accumulation (8.74 ± 0.62%ID/g at 2 h) and similarly stable retention at later time points (8.08 ± 0.51%ID/g at 24 h; 5.25 ± 0.27%ID/g at 7 days). These results confirm that both radiopharmaceuticals effectively target bone tissue and maintain prolonged residence, which is essential for therapeutic irradiation of metastatic lesions and palliation of bone pain.

Despite their similar skeletal kinetics, notable differences emerged in soft-tissue distribution. [^161^Tb]Tb-BPAMD displayed consistently low uptake in major organs such as the liver, spleen, muscle, and blood, with mean values typically below 0.03%ID/g and minimal variability. Kidney uptake was higher at early time points (0.23 ± 0.05%ID/g at 2 h) but declined substantially by day 7 (0.08 ± 0.02%ID/g), indicating efficient clearance. In contrast, [^177^Lu]Lu-BPAMD demonstrated higher soft-tissue activity at early time points, particularly in the kidneys (0.31 ± 0.04%ID/g at 2 h; 0.25 ± 0.06%ID/g at 24 h), as well as slightly higher liver and lung uptake, relative to ^161^Tb.

The target-to-non-target ratios presented in Table 1 support the findings from the biodistribution data and further highlight the distinct performance profiles of [^161^Tb]Tb-BPAMD and [^177^Lu]Lu-BPAMD. Notably, these ratios highlight a key advantage of [^161^Tb]Tb-BPAMD: its enhanced selectivity for bone compared to major organs. Bone/liver ratios are consistently higher for ^161^Tb at all time points, particularly at 24 h (352.7 vs. 161.5), reflecting markedly lower hepatic retention relative to bone. A similar pattern is observed for bone/kidney ratios, where ^161^Tb achieves higher values at each time point, especially at 7 days (66.3 vs. 50.0). This trend corresponds directly with the biodistribution findings, where ^161^Tb exhibited lower soft-tissue uptake, faster non-target clearance, and a pronounced decline in kidney activity over time. These properties indicate a more favorable biodistribution profile, with reduced non-target radiation that may translate into improved therapeutic tolerability. Bone/spleen ratios follow the same trend, with ^161^Tb showing a noticeable advantage after 24 h (468.7 vs. 269.2). These patterns suggest that [^161^Tb]Tb-BPAMD offers more favorable target-to-background ratios over time, with reduced nonskeletal retention and faster washout from soft tissues.

Both [^161^Tb]Tb-BPAMD and [^177^Lu]Lu-BPAMD are highly effective bone-targeting radiopharmaceuticals with largely similar skeletal uptake profiles. However, [^161^Tb]Tb-BPAMD exhibits slightly lower soft-tissue accumulation and faster kidney clearance, which may translate into reduced non-target radiation exposure. These subtle differences are likely attributable to minor variations in the physicochemical properties of the radionuclides, such as ionic radius, coordination kinetics, or in vivo stability of the metal–DOTA complex. In combination with the favorable emission profile of ^161^Tb, including low-energy conversion and Auger electrons that enhance dose deposition in small-scale disease, these findings suggest that [^161^Tb]Tb-BPAMD may offer improved selectivity and an expanded therapeutic potential compared to [^177^Lu]Lu-BPAMD, while both remain highly effective bone-targeting agents.

## 4. Conclusions

[^161^Tb]Tb-BPAMD was successfully prepared with high radiochemical yield, excellent in vitro stability, and strong hydroxyapatite affinity. Its low plasma protein binding, high hydrophilicity, and rapid systemic clearance contribute to a biodistribution profile characterized by selective and stable skeletal uptake with minimal non-target retention. Although both [^161^Tb]Tb-BPAMD and [^177^Lu]Lu-BPAMD demonstrate effective and clinically relevant bone targeting, [^161^Tb]Tb-BPAMD exhibits more selective biodistribution, reflected in lower soft-tissue accumulation and decreasing kidney activity over time. Moreover, while its pharmacokinetic and binding characteristics closely mirror those of [^177^Lu]Lu-BPAMD, [^161^Tb]Tb-BPAMD offers the added therapeutic advantage of high-LET Auger and conversion electrons, potentially enhancing dose delivery to metastatic bone lesions, particularly micrometastases, while reducing systemic toxicity. DFT geometry optimizations of Tb^3+^ and Lu^3+^ BPAMD model complexes support a preferred octa-coordinated binding mode over a hepta-coordinated alternative. Taken together, these findings indicate that [^161^Tb]Tb-BPAMD represents a promising alternative to [^177^Lu]Lu-BPAMD within the class of BPAMD-based radiopharmaceuticals, offering improved selectivity and an expanded therapeutic potential for bone-targeted radionuclide therapy.

## Figures and Tables

**Table 1:** Target to non-target uptake ratio for BPAMD radiolabeled with ^161^Tb and ^177^Lu at 2 h, 24 h, and 7 d in healthy Wistar rats.

Parameter	[^161^Tb]Tb-BPAMD	[^177^Lu]Lu-BPAMD	[^161^Tb]Tb-BPAMD	[^177^Lu]Lu-BPAMD	[^161^Tb]Tb-BPAMD	[^177^Lu]Lu-BPAMD
	2 h		24 h		7 d	
Bone uptake (%ID/g)	8.06 ± 0.61	8.74 ± 0.62	6.70 ± 0.26	8.08 ± 0.51	5.31 ± 0.49	5.25 ± 0.27
Bone/liver ratio	346.1	249.7	352.7	161.5	214.8	131.1
Bone/kidney ratio	34.6	28.2	37.9	32.3	66.3	50.0
Bone/spleen ratio	302.0	249.7	468.7	269.2	196.5	262.3

## Data Availability

The original contributions presented in this study are included in the article. Further inquiries can be directed to the corresponding author.

## Supplementary Materials

The following supporting information can be downloaded at: https://www.mdpi.com/article/10.3390/pharmaceutics18030312/s1, Figure S1: Radio-TLC chromatograms of [^161^Tb]Tb-BPAMD developed in 1M NaOH as mobile phase and ITLC-SG as the stationary phase; Figure S2: Radio-TLC chromatograms of [^161^Tb]TbCl_3_ developed in 1M NaOH as mobile phase and ITLC-SG as the stationary phase; Figure S3: Radio-TLC chromatograms of [^161^Tb]Tb-BPAMD developed in ammonia/ethanol/water (1:10:20, *v*/*v*/*v*) as mobile phase and ITLC-SG as the stationary phase; Figure S4: Radio-TLC chromatograms of [^161^Tb]TbCl_3_ developed in ammonia/ethanol/water (1:10:20, *v*/*v*/*v*) as mobile phase and ITLC-SG as the stationary phase; Figure S5: Proposed ways of coordination for Tb^3+^ and Lu^3+^ with the modified BPAMD ligand (structure **1**: hepta-coordination; structure **2**: octa-coordination); Figure S6: Molecular orbitals with dominant f-character for the octa-coordinated complex 2 of Tb^3+^; Figure S7: Optimized octa-coordinated structure of Tb3+ complex with BPAMD, via one phosphonate group; Scheme S1: Thermodynamic transition from hepta-coordination to octa-coordination (ΔG_Tb_ = −14.40 kcal/mol, ΔG_Lu_ = −8.13 kcal/mol); Table S1: Relative Gibbs free energies (ΔG, kcal/mol) and component energy contributions (kcal/mol) for the investigated Tb^3+^ complexes, expressed with respect to the hepta-coordinated structure **1**; Table S2: Relative Gibbs free energies (ΔG, kcal/mol) and component energy contributions (kcal/mol) for the investigated Lu^3+^ complexes, expressed with respect to the hepta-coordinated structure **1**.
