# Supplementary material for: [161Tb]Tb-BPAMD as a High-Affinity Agent for Skeletal Targeting: Radiochemical and Biodistribution Insights
Source: Pharmaceutics. 2026 Feb 28;18(3):312. doi: 10.3390/pharmaceutics18030312 (PMC13029369; doi:10.3390/pharmaceutics18030312)
Supplement: Supplementary file 1 [file pharmaceutics-18-00312-s001.zip › pharmaceutics-4181913-supplementary.pdf]

SUPPLEMENTARY MATERIALS

[^{161}Tb]Tb-BPAMD as a high-affinity agent for skeletal targeting: radiochemical and biodistribution insights

Magdalena Radović[1], Pavle Sitarica[1], Dragana Stanković[1], Marija Mirković[1], Drina Janković[1], Miloš Marić[1], Marko Perić[1], Sanja Vranješ-Đurić[1], Aleksandar Vukadinović[1]

[1] "VINČA" Institute of Nuclear Sciences - National Institute of the Republic of Serbia, University of Belgrade, Belgrade, Serbia

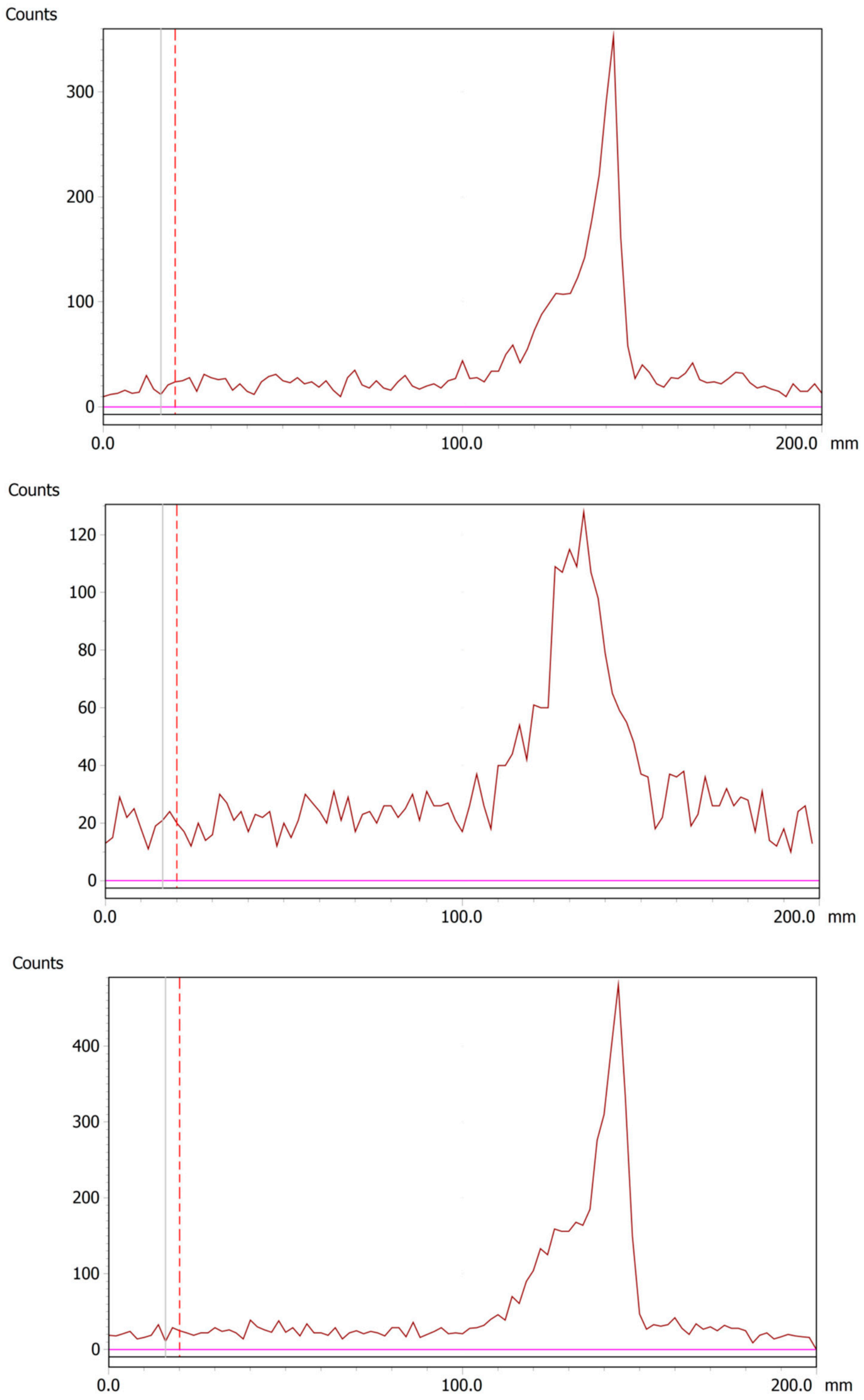

Figure S1: Radio-TLC chromatograms of [^{161}Tb]Tb-BPAMD developed in 1M NaOH as mobile phase and ITLC-SG as the stationary phase.

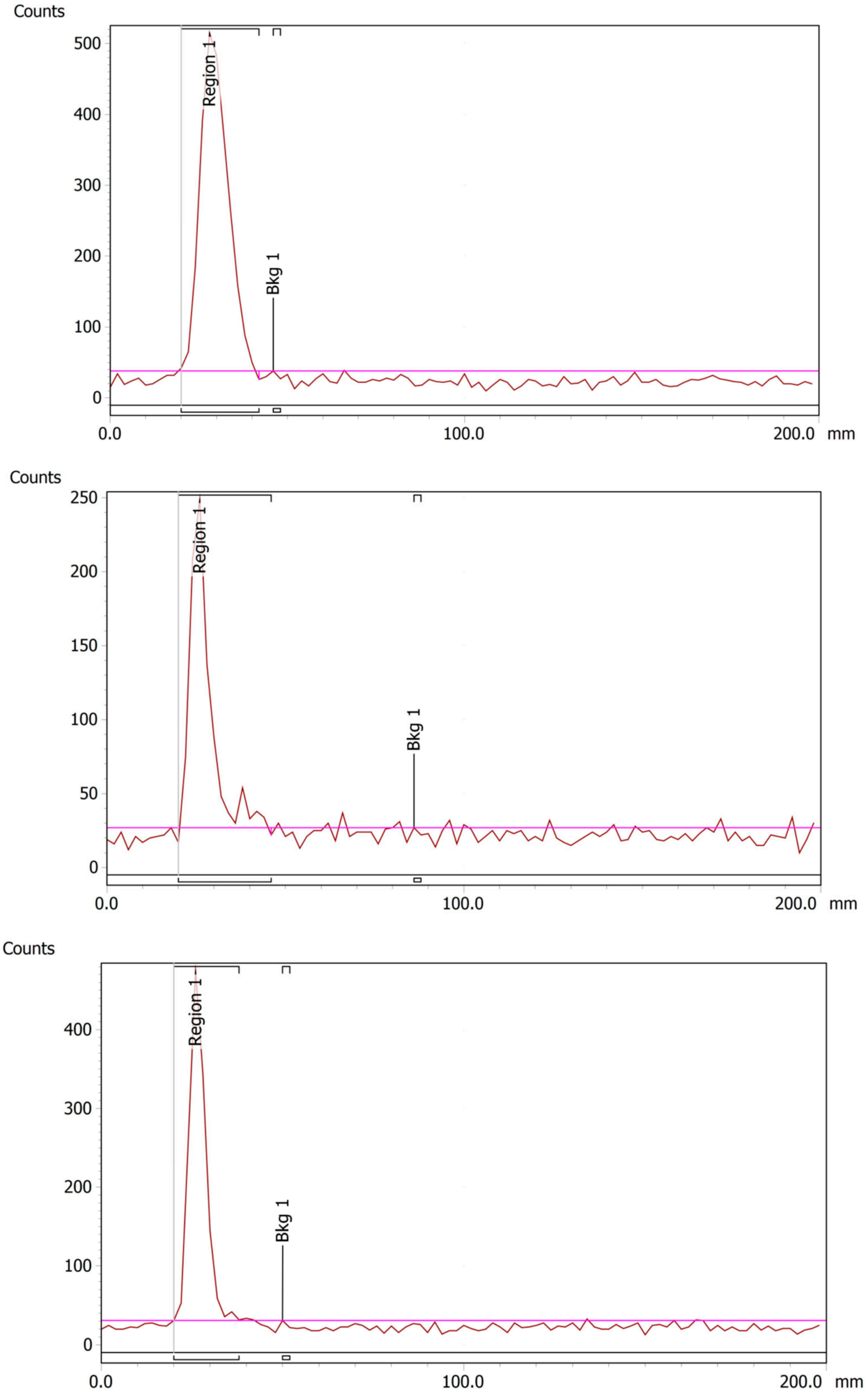

Figure S2: Radio-TLC chromatograms of [^{161}Tb]$TbCl_3$ developed in 1M NaOH as mobile phase and ITLC-SG as the stationary phase.

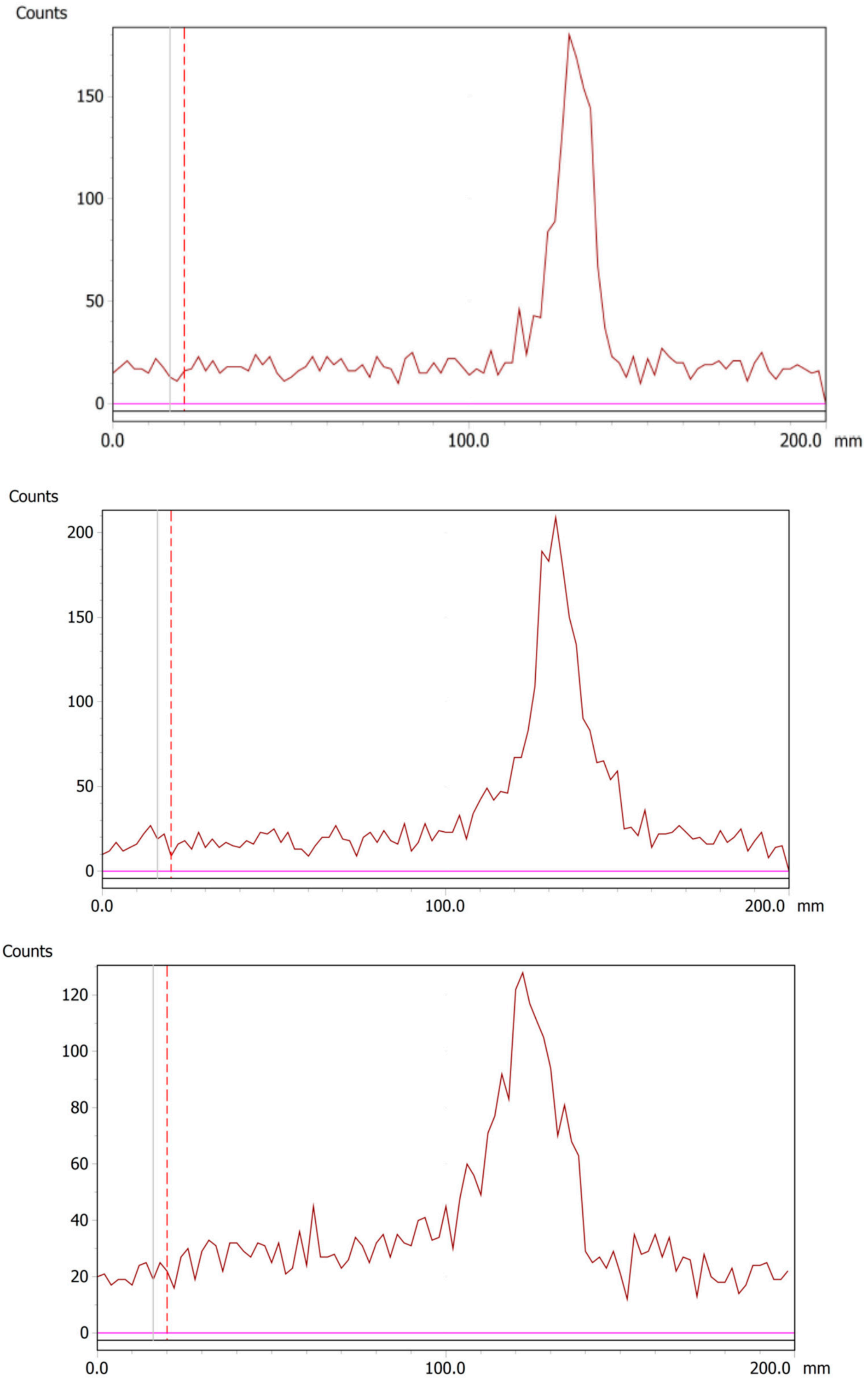

Figure S3: Radio-TLC chromatograms of [^{161}Tb]Tb-BPAMD developed in ammonia/ethanol/water (1:10:20, v/v/v) as mobile phase and ITLC-SG as the stationary phase.

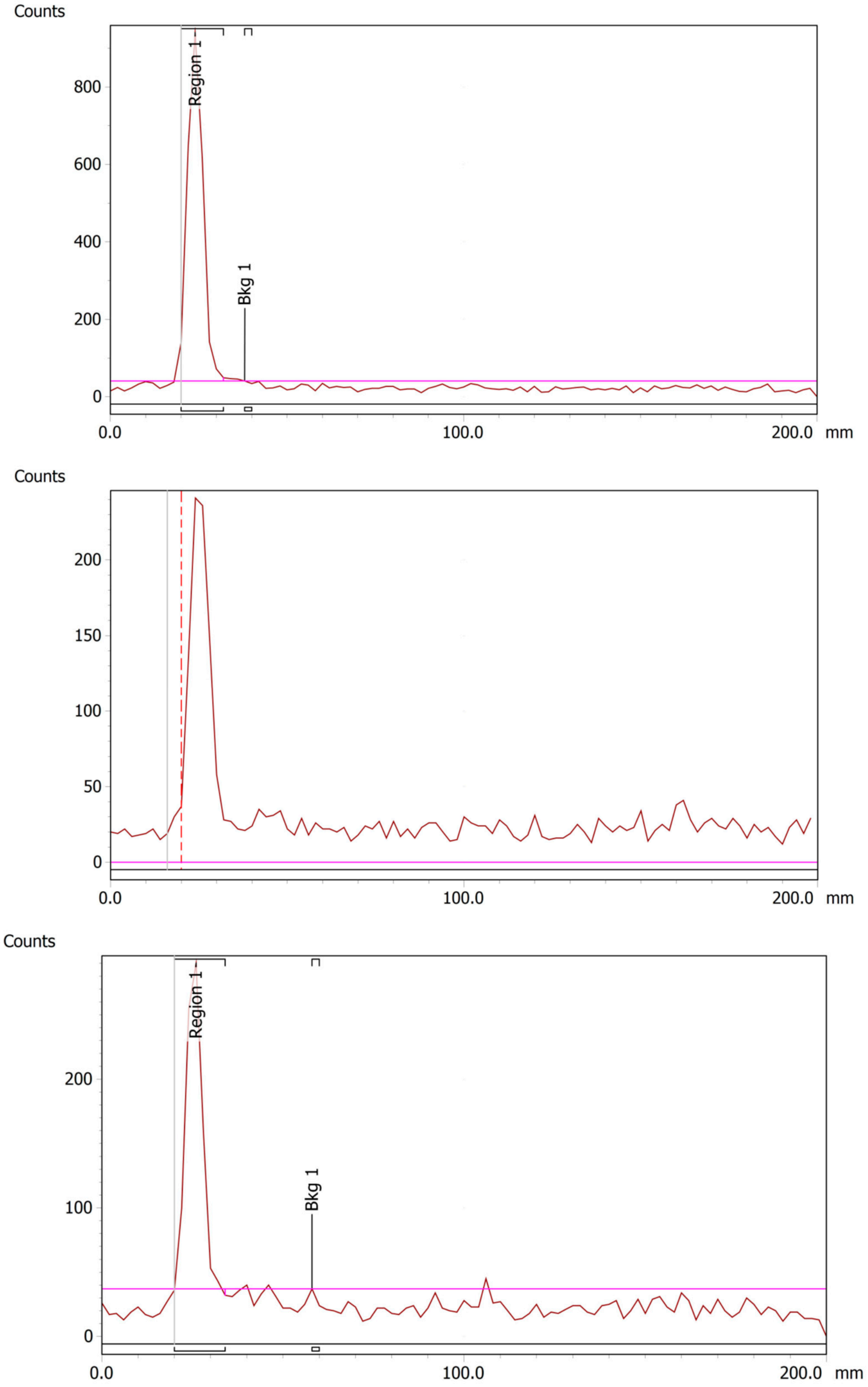

Figure S4: Radio-TLC chromatograms of [^{161}Tb]$TbCl_3$ developed in ammonia/ethanol/water (1:10:20, v/v/v) as mobile phase and ITLC-SG as the stationary phase.

Computational studies

To better understand the coordination chemistry of terbium and lutetium with the BPAMD ligand, two initial structures were analyzed, shown in **Figure S5**.

1 **2**

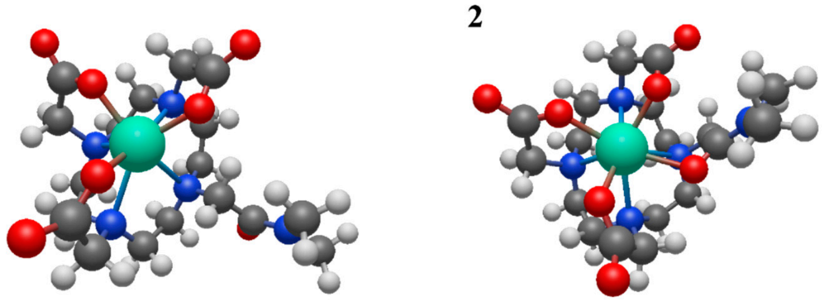

Figure S5. Proposed ways of coordination for Tb^{3+} and Lu^{3+} with the modified BPAMD ligand (structure **1**: hepta-coordination; structure **2**: octa-coordination)

The BPAMD ligand is modified by replacing the phosphonate groups with hydrogen atoms, so the amid nitrogen carries methyl substituents. The BPAMD ligand is modified to avoid possible interactions with phosphonate groups. This allowed us to explore only the coordinative bond with the amid branch. The first structure assumed the hepta-coordination of the chelate ligand, while the second structure assumed the octa-coordination of the modified BPAMD ligand. Geometry optimization of the first proposed structure (hepta-coordinated, structure **1**) proceeded without any alteration to the coordination mode. The absence of imaginary (negative) vibrational frequencies in the resulting structure confirmed that the optimization had converged to a true local energy minimum. Likewise, optimization of the second proposed structure (octa-coordinated structure **2**) maintained its initial coordination geometry without any reorganization during energy minimization. These findings are the same for both coordinated metals, terbium and lutetium. The difference between the Gibbs free energies of complexes 1 and 2 (**Scheme S1**) allows prediction of which structure is more probable. Compared with the second structure, the first structure of terbium has approximately 14 kcal/mol more energy (**Table S1**), and for lutetium, this difference is 8.13 kcal/mol (**Table S2**).

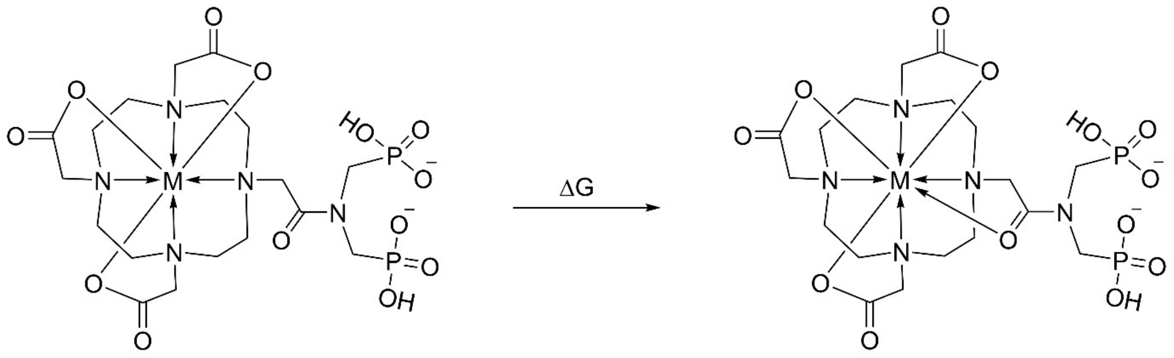

Scheme S1: Thermodynamic transition from hepta-coordination to octa-coordination (ΔG_{Tb} = -14.40 kcal/mol, ΔG_{Lu} = -8.13 kcal/mol)

Table S1. Relative Gibbs free energies (ΔG, kcal/mol) and component energy contributions (kcal/mol) for the investigated Tb^{3+} complexes, expressed with respect to the hepta-coordinated structure **1**.

Complex	**1**	**2**
Nuclear repulsion energy	0	127145.90
Electronic energy	0	-127161.58
$\Delta E_{nuc}+\Delta E_{elec}$	0	-15.68
Enthalpy	0	-14.92
Electronic entropy	0	0.00
Vibrational entropy	0	-0.42
Rotational entropy	0	-0.11
Translational entropy	0	0.00
Total entropy change	0	-0.53
Gibbs free energy change	0	-14.40

Examination of the energy contributions in **Table S1** reveals that nuclear repulsion terms energetically favor the hepta-coordinated structure (structure **1**), whereas electronic stabilization strongly favors the octa-coordinated arrangement (structure **2**). In contrast, entropic contributions overall favor structure **1**, with vibrational entropy providing the dominant positive influence.

In the hepta-coordinated geometry (structure **1**), the uncoordinated amide branch is positioned to maximize its distance from the terbium ion and the other coordinating groups, thereby reducing steric crowding and nuclear repulsion. Transitioning to the octa-coordinated form (structure **2**) brings these groups into closer proximity, which increases nuclear repulsive interactions. At the same time, the additional coordination of the amide branch to terbium results in significant electronic stabilization through enhanced metal–ligand bonding.

The decrease in vibrational entropy upon forming the octa-coordinated complex is consistent with the general behavior of ligands upon metal binding: coordination restricts the ligand's conformational freedom, reducing the number of accessible vibrational modes (such as stretching, bending, and torsional motions). This loss of flexibility lowers the vibrational entropy contribution, providing an entropic penalty that partially offsets the electronic gain.

Lanthanide–ligand interactions are predominantly electrostatic in nature, with Tb^{3+} acting as a hard Lewis acid that preferentially coordinates with hard oxygen donors. The deeply buried 4f electrons contribute minimally to covalent bonding, reinforcing the ionic character of the complex. The molecular orbitals with dominant f-character for structure **2** are shown in **Figure S6**.

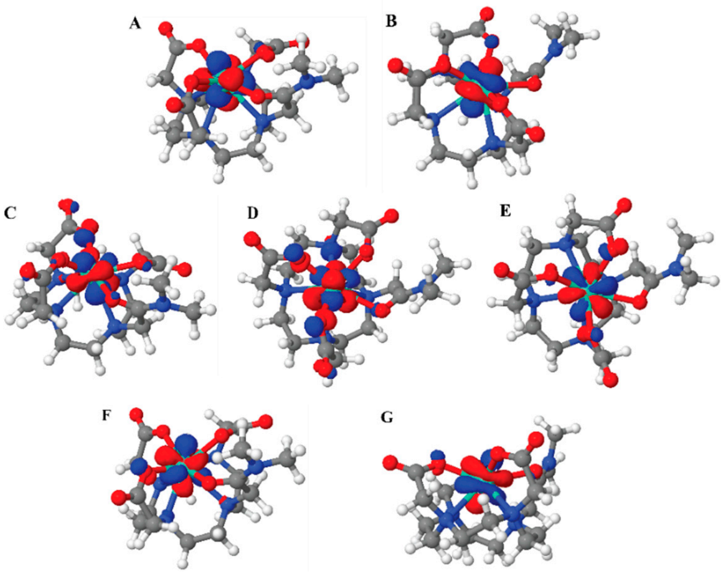

Figure S6. Molecular orbitals with dominant f-character for the octa-coordinated complex 2 of Tb^{3+}.

The preference for higher coordination numbers is also influenced by the ionic radius of the metal ion. As a result of the lanthanide contraction, the reduced ionic radius of Lu^{3+} imposes greater steric constraints, making it less able to accommodate an eighth coordination site than the larger Tb^{3+}. Accordingly, the Gibbs free energy change (ΔG) for the transition from hepta- to octa-coordination in the equivalent Lu^{3+} complexes (**Table S2**) is -8.13 kcal/mol when calculated at the same level of theory, indicating a less pronounced thermodynamic driving force than that observed for Tb^{3+} (ΔG = -14.4 kcal/mol).

Table S2. Relative Gibbs free energies (ΔG, kcal/mol) and component energy contributions (kcal/mol) for the investigated Lu^{3+} complexes, expressed with respect to the hepta-coordinated structure **1**.

Complex	**1**	**2**
Nuclear repulsion energy	0	133275.67
Electronic energy	0	-133284.43
ΔEnuc+ΔEelec	0	-8.77
Enthalpy	0	-7.49
Electronic entropy	0	0.00
Vibrational entropy	0	0.75
Rotational entropy	0	-0.11
Translational entropy	0	0.00
Total entropy change	0	0.64
Gibbs free enthalpy change	0	-8.13

A much lower ΔG for the transition from hepta-coordinated to octa-coordinated complex of lutetium indicates that the larger terbium ion has a stronger tendency towards higher coordination numbers.

However, regardless of the lanthanide used (terbium or lutetium), both metals prefer octa-coordination. The tendency towards the formation of octa-coordinated complexes is even more pronounced if the actual BPAMD ligand is used for coordination. If optimization starts with an octa-coordinated structure (amide branch), energy minimization leads to a structure with the same coordination number eight. However, if geometry optimization starts from the hepta-coordinated initial structure, energy minimization leads to an octa-coordinated structure via one phosphonate group (**Figure S7**).

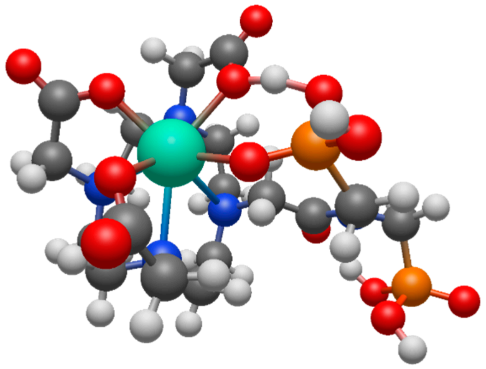

Figure S7. Optimized octa-coordinated structure of Tb3+ complex with BPAMD, via one phosphonate group

DFT calculations showed that the energy difference between octa-coordinated structures via the oxygen of the phosphonate group and via the oxygen of the amid group is negligible. In the case of the Tb^{3+} complex, octa-coordinated structures via the phosphonate group are favored by just 0,4 kcal/mol. In the case of Lu^{3+} complex with BPAMD, the octa-coordinated structure via the phosphonate group is 1,42 kcal/mol higher in energy than the other octa-coordinated structure.
